# DOX-conjugated unimolecular micelles from benzaldehyde-functionalized star copolymer via metal-free ATRP for pH-responsive drug delivery

**DOI:** 10.1080/14686996.2026.2624889

**Published:** 2026-02-02

**Authors:** Wei Ma, Hongyan Yang, Xinyue Ma, Jiao Huang, Li Chen, Atsushi Takahara

**Affiliations:** a Guangxi Key Laboratory of Tumor Immunology and Microenvironmental Regulation, Guilin Medical University, Guilin, PR China; b Guangxi Health Commission Key Laboratory of Tumor Immunology and Receptor-Targeted Drug Basic Research, Guilin Medical University, Guilin, PR China; c School of Public Health, Guilin Medical University, Guilin, PR China; d Research Center for Negative Emission Technologies, Kyushu University, Nishi-Ku, Fukuoka, Japan

**Keywords:** Polymer prodrugs, unimolecular micelles, star copolymers, pH-responsive drug delivery, doxorubicin

## Abstract

The development of polymer prodrugs with well-defined architectures capable of forming stable nanomicelles is important for achieving precise and efficient drug delivery. Compared with conventional linear systems, star-shaped copolymer prodrugs offer enhanced micellar stability owing to their covalently tethered architecture. However, many existing synthetic approaches rely on transition-metal catalyzed polymerization and customized monomers, which limit their sustainability and practical applicability. Herein, we report a pH-responsive doxorubicin (DOX)-conjugated benzaldehyde-functionalized star-shaped copolymer synthesized via an environmentally benign metal-free atom transfer radical polymerization (ATRP) strategy. A hydroxyl-functionalized star-shaped copolymer scaffold was first prepared from readily available monomers and subsequently modified to introduce pendant benzaldehyde groups, enabling DOX conjugation through acid-labile imine linkages. This approach affords well-controlled polymer architectures while avoiding transition-metal residues and the need for pre-functionalized monomers. The resulting DOX-conjugated star-shaped copolymers form unimolecular micelles with high colloidal stability and exhibit pH-triggered drug release under acidic conditions. *In vitro* studies further demonstrate effective cellular internalization and a moderated cytotoxic profile toward human large-cell lung carcinoma (H460) cells, supporting the functional viability of this micellar system as a polymer prodrug delivery platform.

## IMPACT STATEMENT

A metal-free ATRP approach enables the synthesis of a doxorubicin-conjugated star copolymer from readily available monomers, forming stable unimolecular micelles with pH-responsive drug release.

## GRAPHICAL ABSTRACT

**Figure:**
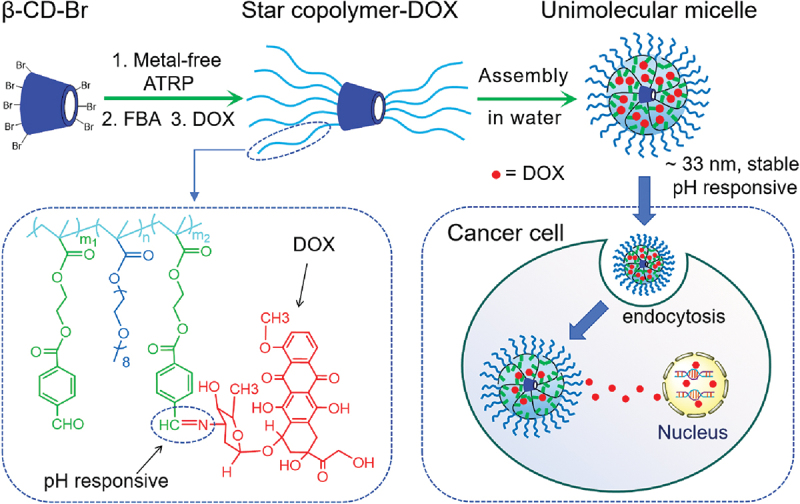

## 1. Introduction

Amphiphilic polymer prodrugs have attracted considerable interest for drug delivery applications owing to their structural tunability, stimulus-responsive drug release, and ability to form nanoscale micellar assemblies [1–3]. Among various external and internal stimuli, pH responsiveness is particularly appealing because of the well-defined pH gradients between normal tissues (pH ≈ 7.4), tumor microenvironments (pH ≈ 6.5–7.0), and acidic intracellular compartments such as endosomes (pH ≈ 5.0–6.0) and lysosomes (pH ≈ 4.5–5.0) [4–6].

pH-responsive polymer prodrugs are commonly designed by incorporating acid-labile linkages, including hydrazone [5,7,8], acetal/ketal [9–11], or imine bonds [12–14], which remain relatively stable under physiological conditions but undergo accelerated cleavage in mildly acidic environments. Upon self-assembly into nanoscale micelles, these systems are generally expected to accumulate in tumor tissues through the enhanced permeability and retention (EPR) effect [15], followed by cellular internalization and acid-triggered drug release in intracellular compartments. This mechanism provides a widely accepted basis for pH-responsive, tumor-associated drug delivery.

Despite significant progress in this area, most reported pH-responsive polymer prodrugs are based on linear architectures, whose micellar assemblies are primarily stabilized by noncovalent interactions. Such assemblies are inherently dynamic and may dissociate upon dilution or in complex biological media due to protein adsorption and other perturbation [16–18]. Although drug molecules may remain covalently bound to polymer chains, disruption of micellar integrity can markedly compromise delivery stability and cellular uptake efficiency [19].

Star-shaped polymers offer an effective strategy to address these limitations associated with linear systems. Owing to their covalently tethered architecture, star-shaped polymers favor the formation of micellar structures with enhanced structural integrity that is less dependent on noncovalent association [20–22]. As a result, such systems typically exhibit superior colloidal stability and can remain intact under conditions that challenge conventional linear micelles [22]. Moreover, the dimensions of micellar structures formed by star-shaped polymers can be modulated by adjusting arm length and composition, which are known to govern nanoscale transport and biodistribution [23–26]. For example, one study reported that micelles with a diameter of ~30 nm exhibited more efficient tumor penetration than larger counterparts (~100 nm) [27]. These features highlight the potential of star-shaped polymers as versatile platforms for constructing stable polymeric nanocarriers with tunable dimensions.

Despite these architectural advantages, the practical realization of star-shaped polymer prodrugs remains strongly dependent on the synthetic methodology, which governs not only structural precision and functional tunability but also scalability, biocompatibility, and environmental sustainability. Although numerous star-shaped polymer drug delivery systems have been reported [22,28–35], their preparation often relies on conventional ATRP employing transition-metal catalysts. Residual metal contamination raises concerns regarding biomedical safety, while catalyst-related waste poses environmental challenges. Furthermore, many existing approaches require the prior synthesis of customized monomers, either pre-functionalized with drug molecules or bearing specific reactive groups [32,36–39]. These additional synthetic steps and purification procedures can significantly limit scalability and practical implementation.

In this work, we address these synthetic and environmental limitations by developing a modular strategy that combines metal-free ATRP [40–42] with post-functionalization to construct a pH-responsive doxorubicin (DOX)-conjugated amphiphilic star copolymer without the use of pre-functionalized monomers. A hydroxyl-functionalized star copolymer scaffold was first prepared via metal-free ATRP and subsequently post-functionalized to introduce pendant benzaldehyde groups, enabling DOX conjugation through acid-labile imine linkages. This strategy simplifies synthesis and enhances structural tunability while avoiding residual transition-metal contamination. The physicochemical properties and *in vitro* biological performance of the resulting DOX-conjugated star copolymer were systematically investigated, demonstrating the formation of stable unimolecular micelles with pH-responsive drug release and measurable anticancer activity *in vitro*.

## 2. Materials and methods

### 2.1. Materials

β-Cyclodextrin (β-CD), 2-bromoisobutyryl bromide (BIBB), 4-formylbenzoic acid (FBA, > 98%), 4-dimethylaminopyridine (DMAP, > 99%), doxorubicin hydrochloride (DOX·HCl), and triethylamine (TEA, > 99%) were purchased from Shanghai Aladdin Reagent Co., Ltd. and used as received. Oligo (ethylene glycol) methyl ether methacrylate (OEGMA, *M*_n_ = 475), 10-phenylphenothiazine (PTH), dichloromethane (DCM), tetrahydrofuran (THF), *N, N*-dimethylformamide (DMF), n-hexane, dimethyl sulfoxide (DMSO), 1-methyl-2-pyrrolidinone (NMP), and *N, N*-dimethylacetamide (DMA) were obtained from Shanghai Macklin Biochemical Technology Co., Ltd. (Shanghai, China). Hydroxyethyl methacrylate (HEMA, 96%) was purchased from Tokyo Chemical Industry Co., Ltd. (TCI). *N, N′*-dicyclohexylcarbodiimide (DCC, > 99%) was obtained from Beijing Innochem Science & Technology Co., Ltd. Dialysis bags (MWCO: 3500 Da) were purchased from Hangzhou Huiying Instrument Business Department (Hangzhou, China). Dulbecco’s Modified Eagle’s Medium (DMEM), Roswell Park Memorial Institute (RPMI) 1640 medium (RPMI-1640), and fetal bovine serum (FBS) were purchased from Energy Chemical Co., (Shanghai, China). Human large cell lung cancer (H460) cells were obtained from Haixing Biosciences Co., Ltd. (Suzhou, China). The cells were cultured in RPMI-1640 medium supplemented with 10% FBS at 37°C in a 5% CO_2_ atmosphere.

### 2.2. Synthesis of star-shaped initiator β-CD-Br

β-CD (2 g, 1.76 mmol) was dissolved in 20 mL of NMP in a round-bottom flask and cooled to 0°C in an ice bath under a nitrogen atmosphere. To this solution, BIBB (13.7 mL, 111 mmol) was added dropwise with continuous stirring. After the addition was complete, the reaction was maintained at 0°C for 2 h. The nitrogen atmosphere was then removed, and the flask was allowed to warm to room temperature, where it was stirred for an additional 24 h. The resulting brown solution was concentrated using rotary evaporation at 65°C for 12 h to yield a brown-yellow syrup-like product. The residue was dissolved in 40 mL of DCM and washed sequentially with saturated aqueous sodium bicarbonate (NaHCO_3_) solution and distilled water. The organic phase was dried over anhydrous sodium sulfate (Na_2_SO_4_), concentrated, and then precipitated into cold n-hexane. This precipitation process was repeated twice. The resulting white solid was dried under vacuum to obtain 3.5 g of the desired product.

### 2.3. Synthesis of star copolymer-OH by metal-free ATRP

Metal-free ATRP of HEMA and OEGMA was conducted using PTH as the catalyst and β-CD-Br as the macroinitiator. In brief, β-CD-Br (0.01 g, 0.0023 mmol), HEMA (0.14 g, 1.08 mmol), and OEGMA (0.85 g, 1.79 mmol) were dissolved in 2.5 mL of DMA in a quartz tube. PTH (0.8 mg, 0.0028 mmol) dissolved in 0.1 mL DMA was then added to the solution. The reaction mixture was degassed by purging with argon for 20 minutes, after which it was irradiated under a 365 nm ultraviolet lamp at room temperature for 5 h. The resulting solution was diluted with THF and precipitated in cold petroleum ether, yielding a sticky product that adhered to the bottom of the flask. The precipitate was dissolved in DMF and transferred into a dialysis bag, which was dialyzed against DMF for 24 h, with the DMF being changed every 3 h. Subsequently, the solution was dialyzed against deionized water for 48 h, with the water being changed every 6 h. The product was obtained after freeze-drying.

### 2.4. Synthesis of the benzaldehyde-functionalized star copolymer (star copolymer-CHO)

Star copolymer-OH (0.66 g) was dissolved in 4 mL of THF in a round-bottom flask. To this solution, FBA (0.21 g, 1.42 mmol) and DMAP (0.027 g, 0.22 mmol) were added under a nitrogen atmosphere, and the mixture was cooled in an ice bath. DCC (0.59 g, 2.84 mmol) dissolved in 3 mL of THF was then added dropwise while stirring magnetically. After the addition, the mixture was removed from the ice bath and allowed to stir at room temperature for 24 h, after which it was filtered. The filtrate was concentrated to yield a pale yellow solid. This solid was dissolved in 7 mL of DMF and transferred into a dialysis bag, which was dialyzed against 100 mL of DMF for 24 h, with the DMF being changed every 3 h. Subsequently, the solution was dialyzed against 500 mL of deionized water for 48 h, with the water being changed every 6 h. The star copolymer (star copolymer-CHO) was obtained after freeze-drying.

### 2.5. Synthesis of DOX conjugated star copolymer (star copolymer-DOX)

Star copolymer-CHO (0.1 g) was dissolved in 4 mL of ultra-dry DMF. DOX·HCl (98.6 mg, 0.17 mmol) and TEA(118 µL, 0.85 mmol) were dissolved in 2.5 mL of ultra-dry DMSO and stirred in the dark at room temperature for 5 h. The resulting DOX solution was then added dropwise into the star copolymer-CHO solution under stirring. After 48 h of reaction under vigorous stirring in the dark at room temperature, the mixture was transferred into a dialysis bag and dialyzed against 200 mL of DMF for 48 h (with the DMF being changed every 6 h) to remove unreacted DOX. The process was monitored by ultraviolet-visible (UV-Vis) spectroscopy until no characteristic DOX absorption peaks were detected in the dialysate, confirming complete removal of physically adsorbed drug molecules. The solution was subsequently dialyzed against 500 mL of deionized water for 48 h (with the water being changed every 6 h) to remove DMF. The reddish-brown star copolymer-DOX was obtained after freeze-drying.

### 2.6. Fabrication of micelles

Star copolymer-CHO (20 mg) and star copolymer-DOX (20 mg) were separately dissolved in 2 mL of THF to obtain solutions with a concentration of 10 mg/mL. Then, 10 mL of deionized water was added dropwise to each solution under continuous stirring. The mixtures were left uncovered at room temperature for 3 days to allow for the complete evaporation of THF. The volume of evaporated water was determined by measuring the weight of the solutions, and an equivalent amount of water was added to restore the original volume, resulting in micelles with a final concentration of 2 mg/mL. To prepare micelles of other concentrations (1.0, 0.5, and 0.1 mg/mL), the solutions were diluted with deionized water.

### 2.7. General characterization

The ^1^H NMR spectra were recorded on a Bruker Ascend 500 MHz spectrometer (Bruker, Germany) using CDCl_3_ as the solvent. UV-Vis absorption spectra were obtained using a UV-2600i spectrophotometer (Shimadzu, Japan). Fourier transform infrared (FT-IR) spectra were obtained on a Jasco-FTIR-4X system (JASCO, Japan) using the KBr disk method. X-ray photoelectron spectroscopy (XPS) was performed by PHI GENESIS 500 (Physical Electronics, USA) with a monochromatic Al Kα X-ray source. The molecular weights of the polymers were estimated by gel permeation chromatography (GPC) on a Shimadzu LC-40D system (Shimadzu, Japan), equipped with a refractive index detector (RID-20A) and a Shodex KF-805 L chromatographic column. DMF was used as the eluent, and molecular weight calibration was performed using narrowly distributed PMMA standards. The particle size and zeta potential of the drug-loaded and blank polymeric micelles were measured by dynamic light scattering (DLS) on Malvern Zetasizer Pro (Malvern Panalytical, UK). Transmission electron microscopy (TEM) images were obtained using a JEOL-1400 Flash microscope (JEOL, Japan). The TEM sample was prepared by dropwise addition of 20 μL of micelle solution (0.1 mg/mL) on a carbon-coated copper grid, followed by negative staining using 2% phosphotungstic acid solution.

### 2.8. Drug loading content and in vitro drug release

A total of 3.5 mg of lyophilized star copolymer-DOX powder was dissolved in 5 mL of DMF. The absorbance of the solution at 480 nm was measured using UV-Vis spectrophotometry. The DOX concentration in the solution was determined by referencing a standard calibration curve of DOX in DMF. The drug loading content (DLC) was then calculated according to formula (1).

(1) $$\mathrm{DLC}(\mathrm{wt}\%)=\frac{\mathrm{weight}\;\mathrm{of}\;\mathrm{DOX}\;\mathrm{in}\;\mathrm{star}\;\mathrm{copolymer}-\mathrm{DOX}}{\mathrm{weight}\;\mathrm{of}\;\mathrm{star}\;\mathrm{copolymer}-\mathrm{DOX}}\times 100\%$$

The *in vitro* release behavior of DOX from the micelles at different pH values was investigated using the dialysis method. Briefly, 0.2 mL of star copolymer-DOX micelle solution (1.5 mg/mL) was placed in a dialysis tube (molecular weight cutoff: 3500 Da) and dialyzed against 40 mL of phosphate-buffered saline with various pH (pH = 7.4, 6.5, 5.0) in a shaking incubator set at 37°C and 120 rpm in the dark. At specified time intervals, 3 mL of the dialysis medium was withdrawn and replaced with an equal volume of fresh PBS at the corresponding pH. The absorbance of the collected dialysate was measured at 480 nm, and the DOX concentration was determined using calibration curves prepared with DOX solutions in PBS buffers of the respective pH. The cumulative DOX released from micelles was calculated using formula (2), where Ve represents the sampling volume at each time point (3 mL), V_0_ denotes the total buffer volume (40 mL), C_i_ is the DOX concentration in the dialysis medium collected during the i-th extraction, and n represents the total number of samples.

(2) $$\mathrm{Cumulative}\;\mathrm{release}(\%)=\frac{V_{e}\times \sum_{i=1}^{n-1}C_{i}+V_{0}\;\times \;C_{n}}{\mathrm{weight}\;\mathrm{of}\;\mathrm{DOX}\;\mathrm{in}\;\mathrm{the}\;\mathrm{micelles}}\times 100\%$$

### 2.9. Stability of the star copolymer-DOX micelles

Stability of the star copolymer-DOX micelles was assessed by monitoring changes in particle size and zeta potential over time. The star copolymer-DOX micelles (0.5 mg/mL) were stored in dark at room temperature for a period of 10 days. Particle size and zeta potential were measured at regular intervals using DLS. Additionally, the particle size of the micelles was also evaluated in different media, including PBS, DMEM, and RPMI-1640 medium, all at 37°C.

### 2.10. In vitro cytotoxicity assay

H460 cells were seeded in 96-well plates at a density of 5 × 10^3^ cells per well and incubated at 37°C in a 5% CO_2_ atmosphere for 24 h. After this incubation, the culture medium was removed, and 100 µL of fresh medium containing varying concentrations of free DOX, star copolymer-CHO micelles, or star copolymer-DOX micelles was added to each well. The cells were then incubated for an additional 48 h. Following this, 10 µL of CCK-8 solution was added to each well, and the plates were further incubated for 3 h. Absorbance at 450 nm was measured using an Infinite M PLEX microplate reader (Tecan, Durham, U.S.A.) to determine cell viability.

### 2.11. Flow cytometric analyses of cells after treatment with free DOX and the star copolymer-DOX micelles

H460 cells were seeded into 6-well plates at a density of 2.5 × 10^5^ cells per well in 2 mL of complete RPMI-1640 medium and incubated at 37°C in a 5% CO_2_ humidified atmosphere for 24 h. After this incubation, the medium was replaced with 2 mL of fresh medium containing either free DOX or star copolymer-DOX micelles (with equivalent DOX concentration of 8 µg/mL). The cells were then incubated for an additional 4 h. Following incubation, the cells were washed twice with PBS, and 0.25% trypsin solution was added to digest the cells. The resulting cell suspensions were collected by centrifugation, and the pellet was resuspended in 200 µL of PBS for analysis by flow cytometry (BD Biosciences Accuri C6). Cells treated with PBS served as a control. Flow cytometry experiments were independently performed using three biological replicates (*n* = 3), and data processing was carried out by averaging the measured fluorescence intensities with standard deviation.

### 2.12. Confocal laser scanning microscopy (CLSM) visualization

To further investigate the cellular uptake process of the star copolymer-DOX micelles, the treated cells were visualized by confocal laser scanning microscopy. In brief, H460 cells were seeded onto 12 mm coverslips in 24-well plates at a density of 1 × 10^5^ cells per well in 1 mL of complete RPMI-1640 medium and incubated at 37°C in a 5% CO_2_ humidified atmosphere for 24 hours. The medium was then replaced with 1 mL of fresh medium containing either free DOX or star copolymer-DOX micelles (with an equivalent DOX concentration of 8 µg/mL), and the cells were incubated for specific time points. After incubation, the cells were washed three times with PBS and fixed with 4% paraformaldehyde for 30 minutes. The coverslips were mounted onto microscope slides with a drop of DAPI containing antifade mounting medium. Cellular uptake was visualized using CLSM (Carl Zeiss LSM 710). CLSM experiments were independently repeated at least three times using separately prepared cell samples, and representative fluorescence images are shown.

### 2.13. Statistical analysis

Statistical analysis was performed using one-way analysis of variance (ANOVA), followed by post hoc multiple comparison tests. Data are presented as mean ± SD (*n* = 3).

## 3. Results and discussion

### 3.1. Synthesis and characterization of the star copolymers and DOX-prodrug

**Figure 1.**
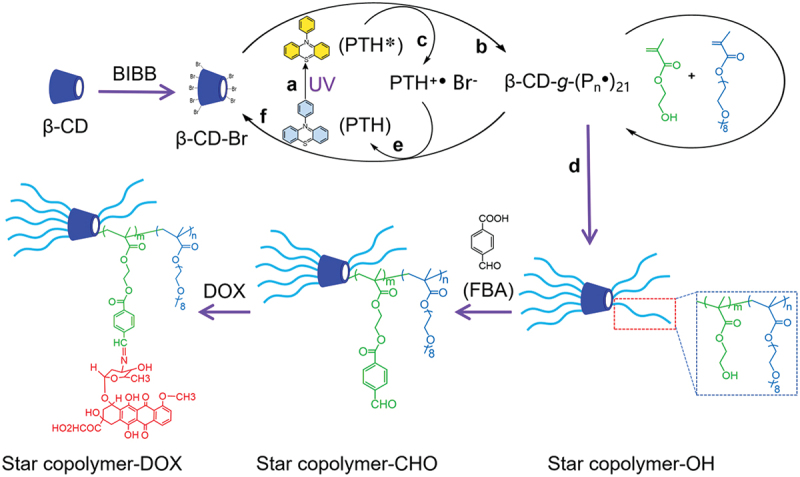
Illustration of the synthetic route for the star copolymer-DOX prodrug. The synthesis mechanism of star copolymer-OH via metal-free ATRP involves the following steps: a. UV light excites PTH to form the excited state PTH*; b. The initiator reacts with PTH*, generating active free radical; c. PTH* transfers electrons to Br, generating PTH^+•^; d. The free radical reacts with monomers (OEGMA and HEMA), leading to chain growth; e. PTH^+•^ returns to its ground state; f. The free radical is deactivated to dormant state. P_n_ = poly(HEMA-*co*-OEGMA).

**Figure 2.**
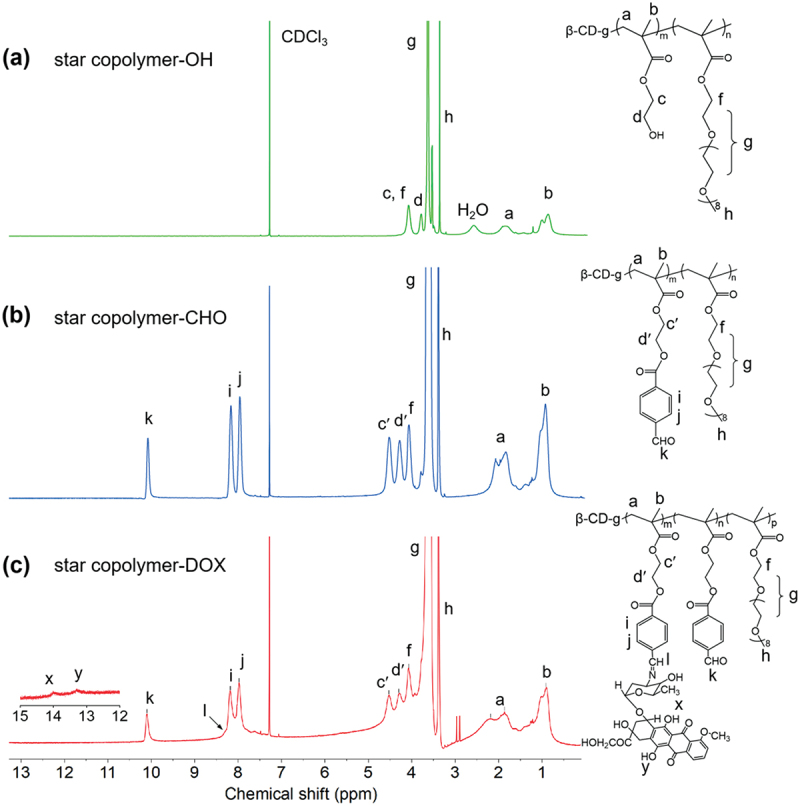
^1^H NMR spectra and chemical structures of (a) the star copolymer-OH, (b) the star copolymer-CHO, and (c) the star copolymer-DOX prodrug.

The chemical structure and synthetic route for the star copolymer-DOX is shown in Figure 1. Firstly, a multi-arm macroinitiator (β-CD-Br) was synthesized by reacting β-CD with BIBB. Then, OH-functionalized star copolymer (star copolymer-OH) was obtained via metal-free ATRP of OEGMA and HEMA on β-CD-Br with PTH [43] as the photocatalyst. Subsequently, star copolymer-CHO was obtained by post-modification with FBA. Last, DOX was conjugated to star copolymer-CHO through imine bond formation to generate the star copolymer-DOX. ^1^H NMR spectra of the star copolymer-OH, star copolymer-CHO, and star copolymer-DOX are displayed in Figures 2a, 2b, and 2c, respectively.

**Figure 3.**
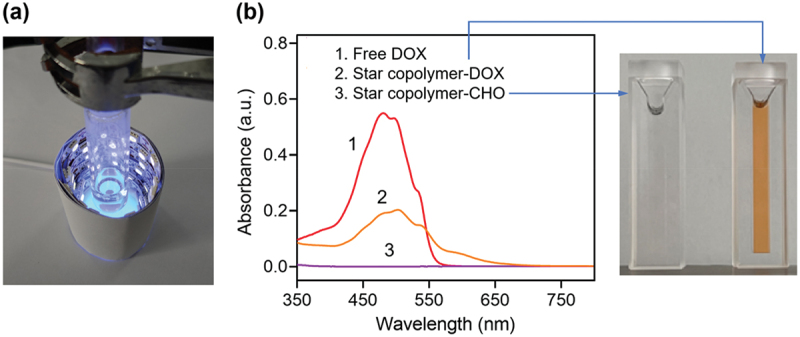
(a) A photograph of the experimental setup used for the metal-free ATRP; (b) UV-vis absorption of free DOX, star copolymer-DOX, and star copolymer-CHO, along with the photograph of the DMF solutions.

Furthermore, the polymerization mechanism of star copolymer-OH via metal-free ATRP is schematically illustrated in the steps of a-f in Figure 1. Upon UV irradiation, PTH is photoexcited to its singlet state (PTH*), which subsequently transfers an electron to the bromine-terminated chain end of the initiator, thereby generating active radicals that can initiate polymerization. Meanwhile, PTH* is converted to an oxidized species (PTH^+ •^), which can reversibly deactivate the propagating radicals back to their dormant form. This reversible activation-deactivation equilibrium maintains the radical concentration at a low level, which is essential for achieving controlled/living radical polymerization. The experimental setup used for the metal-free ATRP is shown in Figure 3a, where a quartz reaction vessel was employed to ensure efficient UV light transmission.

The chemical structure of star copolymer-OH was confirmed by ^1^H NMR (Figure 2a). Resonances at 3.4 ppm (peak h) and 3.6 ppm (peak g) correspond to the methoxy protons and -CH_2_-CH_2_-O- repeating units of OEGMA, while signal at 3.8 ppm (peak d) arises from HEMA methylene protons. These assignments confirm the grafting of poly(HEMA-*co*-OEGMA) chains onto the β-CD core. Integration analysis gave a HEMA : OEGMA ratio of 0.9:1 (Supporting Information), which is higher than the feed ratio (0.6:1), suggesting that OEGMA exhibits lower polymerization activity than HEMA, presumably due to the steric hindrance arising from its longer side chain.

Subsequent modification with FBA yielded the benzaldehyde-functionalized star copolymer. ^1^H NMR (Figure 2b) showed attenuation of the HEMA methylene signal at 3.8 ppm, and appearance of new peaks at 10.1 ppm (aldehyde proton, peak k) and 8.0–8.2 ppm (aromatic protons, peaks j and i). Peaks at 4.3 and 4.5 ppm (peaks d′ and c′) corresponded to methylene groups of FBA-modified HEMA. These spectral changes collectively confirm the transformation of hydroxyl groups to benzaldehyde functionalities, with a conversion ratio of approximately 99% (Supporting Information). The molecular characteristics of the star copolymers at different synthetic stages were further examined by GPC analysis, with detailed data provided in the Supporting Information (Figure S1).

The pendant benzaldehyde groups on the star copolymer were subsequently reacted with the amino groups of DOX, yielding acid-labile imine linkages. ^1^H NMR spectra of the resulting star copolymer-DOX (Figure 2c) showed characteristic DOX signals at 13.3 and 14.0 ppm, while the intensity of the aldehyde resonance at 10.1 ppm decreased relative to the benzene signals (8.0–8.2 ppm), consistent with imine formation. The expected imine resonance (typically observed at 8.1–8.4 ppm [44]) was not clearly resolved, likely because of overlap with the aromatic resonances, which limited the use of ^1^H NMR for quantitative determination of the aldehyde-to-DOX conversion. UV-Vis spectroscopy further verified the successful conjugation of DOX. The star copolymer-CHO solution was colorless and exhibited no absorption across the entire wavelength range, whereas the star copolymer-DOX solution appeared orange and displayed a characteristic absorption band of DOX (Figure 3b). Accordingly, the extent of DOX conjugation was evaluated by UV-Vis spectroscopy, giving a drug loading content (DLC) of approximately 20%. It is worth noting that steric hindrance associated with the densely grafted star polymer architecture is likely a contributing factor to the relatively moderate drug loading level.

FTIR, XPS, and GPC analyses provided additional supporting evidence for DOX conjugation. GPC analysis (Figure S1, Supporting Information) showed an apparent increase in molecular weight after DOX conjugation. Compared with the star copolymer-CHO, the star copolymer-DOX exhibited new spectral features in the 1600–1700 cm^−1^ region in the FTIR spectra as well as a discernible N _1s_ signal at ~399 eV in the XPS spectra (Figures S2 and S3, Supporting Information), which are suggestive of the introduction of a nitrogen-containing chemical environment expected for imine-containing conjugates.

### 3.2. Formation and stability of the unimolecular micelles

**Figure 4.**
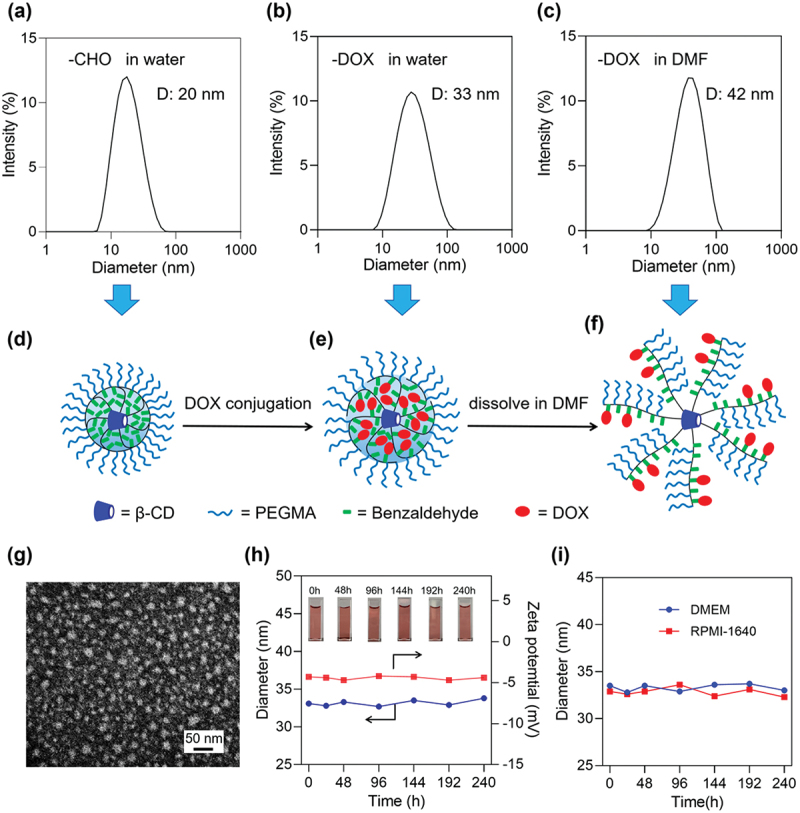
DLS data of the star copolymer-CHO in water (a), the star copolymer-DOX in water (b), and the star copolymer-DOX in DMF (c); schematic structural models of the star copolymer-CHO in water (d), the star copolymer-DOX in water (e), and the star copolymer-DOX in DMF (f); (g) TEM image of the star copoymer-DOX micelles in dehydrated state; (h) particle size and zeta potential stability of the star copolymer-DOX micelles in PBS; (i) particle size stability of the star copolymer-DOX micelles in DMEM and RPMI-1640.

Owing to their amphiphilic nature, both star copolymer-CHO and star copolymer-DOX readily self-assemble into micellar structures in aqueous media. DLS measurements showed that the star copolymer-CHO micelles possessed an average hydrodynamic diameter of approximately 20 nm (Figure 4a). Upon DOX conjugation, the micellar diameter increased to about 33 nm (Figure 4b), indicating successful drug incorporation. TEM image further revealed that the star copolymer-DOX micelles exhibited a spherical morphology with particle size around 20 nm (Figure 4g). The smaller sizes observed by TEM compared with DLS results can be attributed to the dehydrated state of the samples under vacuum condition.

To clarify whether the micelles were unimolecular or multimolecular assemblies, DLS measurements were further conducted in DMF, a non-selective solvent for the star copolymer-DOX, in which the polymer chains are well solvated and aggregation-driven self-assembly is effectively suppressed. The hydrodynamic diameter of the star copolymer-DOX prodrug measured in DMF (~42 nm, Figure 4c) was comparable to that observed in water (~33 nm), suggesting that the assemblies formed in aqueous media are predominantly unimolecular micelles. In contrast, if multimolecular aggregation had occurred, a substantially larger hydrodynamic diameter would be expected in water than in DMF due to solvent-induced dissociation of noncovalent assemblies.

Figures 4d-f schematically illustrate the proposed structural models of the star copolymer-CHO micelles in water, the star copolymer-DOX micelles in water, and the star copolymer-DOX in DMF, respectively. The slightly larger apparent hydrodynamic diameter of the star copolymer-DOX observed in DMF compared with that in water can be rationalized by differences in polymer chain conformation. In aqueous media, the amphiphilic star copolymers adopt a compact core-shell structure, in which the hydrophobic segments collapse to form dense micellar cores (Figure 4d-e). In contrast, in DMF both the hydrophilic poly (OEGMA) segments and the hydrophobic domains are effectively solvated, leading to an expanded chain conformation that results in a larger hydrodynamic diameter (Figure 4f).

Because the star copolymer was synthesized via a controlled/living radical polymerization, its molecular weight can be regulated through adjustment of the polymerization conditions. Variations in molecular weight are generally expected to influence the dimensions of micellar structures. Beyond molecular weight control, the relative content of the pendant monomers in the star copolymer arms can also be tuned by the monomer feed ratio during synthesis. This compositional parameter is anticipated to affect the availability of benzaldehyde functionalities for DOX conjugation and may consequently influence the drug loading level as well as the size of the resulting micelles. However, the present study focuses on establishing a robust unimolecular micelle platform and evaluating its pH-responsive drug delivery behavior, rather than micelle size modulation.

The colloidal stability of the star copolymer-DOX micelles was evaluated under physiologically relevant conditions. As shown in Figure 4h, both the hydrodynamic diameter and zeta potential remained essentially constant over 240 h in PBS, indicating excellent long-term stability in buffered solution. Similarly, the particle size exhibited negligible changes in cell culture media (DMEM and RPMI-1640, Figure 4i), highlighting the effectiveness of the hydrophilic poly(OEGMA)-based corona in maintaining micellar stability even under complex ionic conditions.

Given that nanocarriers inevitably experience substantial dilution in blood stream upon intravenous administration, maintaining structural integrity across a wide range of concentration is critical for advanced drug delivery systems. Dilution experiments confirmed this robustness: the hydrodynamic diameter of the star copolymer-DOX micelles remained essentially constant as the polymer concentration decreased from 2.0 to 0.1 mg/mL (Figure S4, Supporting Information). This concentration‐independent behavior aligns with the characteristics of unimolecular micelles, whose integrity is ensured by the covalent linkages. The ability of the star copolymer-DOX micelles to maintain structural integrity across a broad dilution range highlights their potential for reliable drug delivery.

### 3.3. In vitro DOX release and cytotoxicity

**Figure 5.**
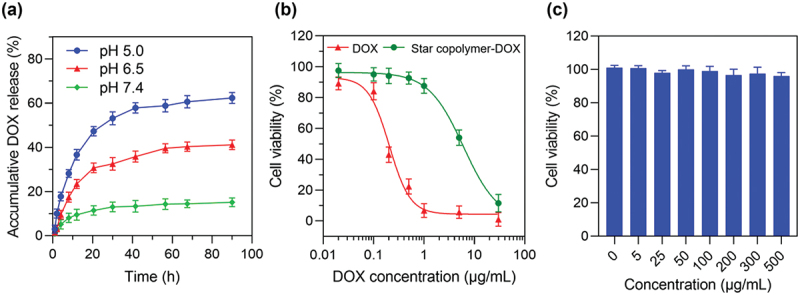
(a) DOX release profile from the star copolymer-DOX micelles at 37°C under different pH (5.0, 6.5, and 7.4); (b) cytotoxicity of free DOX and the star copolymer-DOX micelles against H460 cells after 48 h incubation; (C) cell viability of H460 cells treated with the star copolymer-CHO at different concentrations for 48 h incubation.

The *in vitro* release of DOX from the star copolymer-DOX micelles was investigated using a dialysis method under three different pH conditions: pH 7.4 to simulate the physiological plasma environment, pH 6.5 to represent the mildly acidic tumor microenvironment, and pH 5.0 to approximate the endo/lysosomal compartments of tumor cells. The cumulative release of DOX into the external medium was quantified by UV-Vis spectroscopy. Figure 5a shows that the DOX release exhibited a pronounced pH-dependent behavior. The drug release at lower pH was much faster than that at higher pH. At pH 7.4, only 15.1% of DOX was released over 90 h, indicating high stability under physiological conditions. In contrast, the release amount increased to 41.1% at pH 6.5 and 62.3% at pH 5.0, respectively, over the same period. The pH-responsive drug release was attributed to the acid labile imine linkage between the star copolymer and the drug molecule.

The cytotoxicity of the prodrug micelles was evaluated in H460 cells. Both free DOX and the star copolymer-DOX micelles exhibited a clear concentration-dependent inhibition of cell viability (Figure 5b). However, at equivalent DOX concentrations, cells treated with the star copolymer-DOX micelles consistently exhibited higher viability than those treated with free DOX. Quantitative analysis (Figure S5, Supporting Information) showed that the half-maximal inhibitory concentration (IC_5__0_) value of the star copolymer-DOX micelles, based on DOX-equivalent concentration, was 6.1 µg mL^−1^, substantially higher than that of free DOX (0.21 µg mL^−1^). These results indicate that, relative to free DOX, the star copolymer-DOX micelles exhibit reduced cytotoxicity while retaining measurable anticancer activity. The difference in cytotoxicity likely arises from distinct cellular uptake behaviors, which were further investigated through flow cytometry and confocal microscopy, as discussed below. Importantly, the carrier itself (star polymer-CHO) exhibited negligible cytotoxicity, maintaining >95% cell viability across all tested concentrations (0–500 µg mL^−1^, Figure 5c), confirming that the observed cytotoxicity originated from DOX release rather than the carrier material.

### 3.4. In vitro cellular uptake

**Figure 6.**
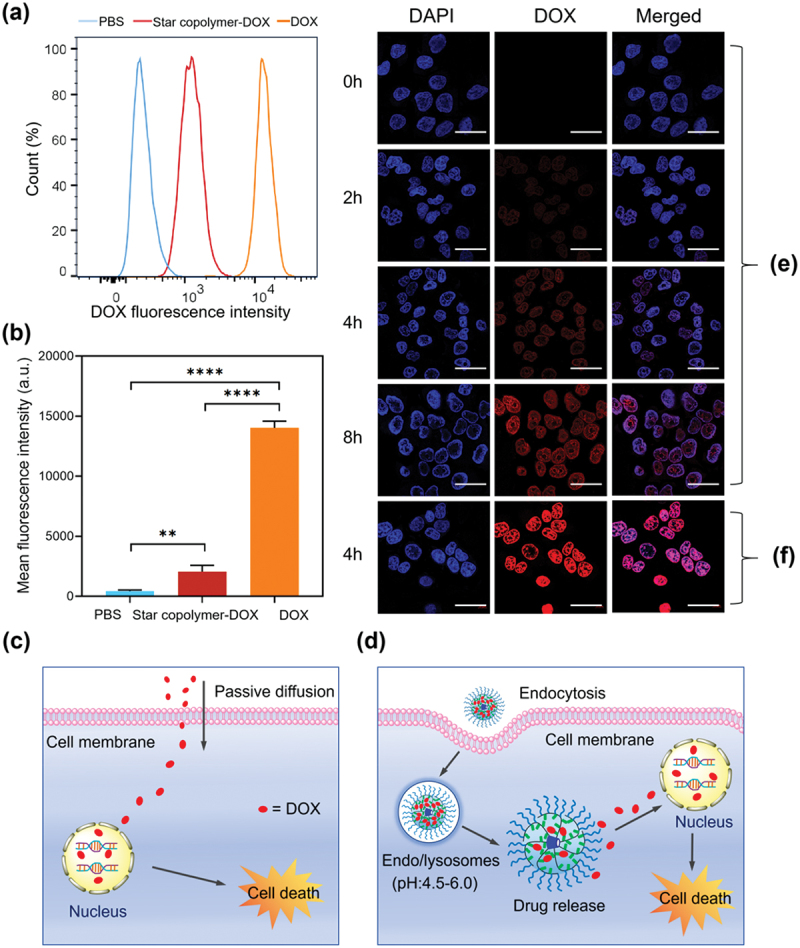
Flow cytometry analysis of H460 cells treated with PBS, the star copolymer-DOX, or free DOX: (a) representative histograms and (b) corresponding mean fluorescence intensity (*n* = 3) (c) schematic illustration of the proposed process by which free DOX enter cells by passive diffusion and localize in nuclei to induce cell death. (d) Schematic illustration of the proposed process by which the star copolymer-DOX micelles undergo endocytosis into cancer cells, release the drug intracellularly in acidic compartments, and ultimately induce cell death. Fluorescence images of H460 cells incubated with the star copolymer-DOX micelles for different incubation times (e) and free DOX for 4 h (f). For each panel, images from left to right show cell nuclei stained by DAPI (blue), DOX fluorescence (red), and overlays of the blue and red images (merged). The scale bars correspond to 50 µm for all images.

Cellular uptake was evaluated by flow cytometry. Figures 6a and 6b present the flow cytometry histograms and the corresponding mean fluorescence intensities of H460 cells treated with PBS, free DOX, and the star copolymer-DOX micelles, respectively. Both the free DOX and the star copolymer-DOX micelles showed significantly higher fluorescence signals than the PBS control, confirming efficient cellular internalization in both cases. However, the fluorescence intensity of cells treated with the star copolymer-DOX micelles was noticeably lower than that of free DOX, suggesting a reduced apparent cellular uptake, which is likely associated with differences in their internalization pathways. Free DOX, owing to its lipophilic and cationic nature, can readily penetrates the cell membrane via passive diffusion (Figure 6c), whereas the micelles are predominantly internalized through endocytosis (Figure 6d), which is intrinsically less rapid than passive diffusion. In addition, the poly(OEGMA)-derived (PEG-like) corona further retards micelle internalization by imparting a ‘stealth’ effect that suppresses nonspecific interactions with cells in the absence of targeting ligands [45].

****p < 0.0001; **p < 0.01.

Furthermore, the intracellular trafficking of DOX was examined using CLSM. Figures 6e and 6f display the fluorescence images of H460 cells incubated with the star copolymer-DOX micelles and free DOX, respectively. The nuclear fluorescence intensity of DOX in cells treated with the star copolymer-DOX micelles gradually increased with incubation time (Figure 6e), suggesting progressive nuclear localization of DOX. However, under the same incubation period of 4 h, the nuclear fluorescence signal in cells treated with the star copolymer-DOX micelles was noticeably weaker than that observed in cells treated with free DOX (Figure 6f). This observation suggests that DOX delivered via the star copolymer-DOX micelles reaches the cell nucleus at a slower rate than free DOX. Such behavior is likely attributable to the reduced apparent cellular uptake of the star copolymer-DOX micelles, together with the requirement for intracellular DOX release from the star copolymer-DOX micelles prior to nuclear diffusion.

Although free DOX can rapidly enter cells and accumulate in the nucleus, it exhibits limited selectivity and can readily diffuse across normal vasculature, which is widely associated with nonspecific distribution in healthy tissues and systemic toxicity. In contrast, the star copolymer-DOX micelles, as a class of polymeric micellar carriers, share nanoscale features that are generally considered capable of facilitating passive tumor accumulation via the EPR effect; this property may help improve tumor-associated distribution while reducing off-target exposure, even though cellular internalization and nuclear accumulation occur more gradually than for free DOX.

### 3.5. Comprehensive property comparison

**Figure 7.**
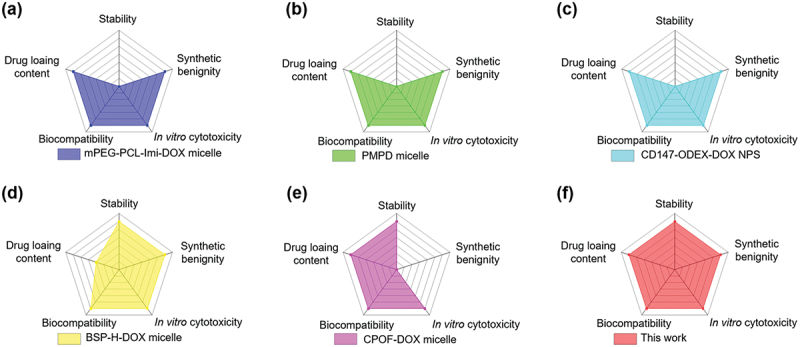
Comparison of reported imine-based DOX prodrug micelles and the present star copolymer-DOX micelles in terms of stability, drug loading content, biocompatibility, *in vitro* cytotoxicity, and synthetic benignity. (a) mPEG-PCL-Imi-DOX micelles [46]; (b) PMPD micelles [13]; (c) CD147-ODEX-DOX NPS [47]; (d) BSP-H-DOX micelles [48]; (e) CPOF-DOX micelles [32]; (f) this work. Scoring criteria are provided in the Supporting Information.

For comparative evaluation, the star copolymer-DOX micelles were benchmarked against several reported imine-linked DOX prodrug nanocarriers in terms of stability, drug-loading content, biocompatibility, *in vitro* cytotoxicity, and synthetic benignity (Figure 7). Although many of these systems exhibit satisfactory *in vitro* cytotoxic performance and acceptable biocompatibility, their stability and preparation routes vary substantially. Linear amphiphilic prodrugs, such as mPEG-PCL-Imi-DOX [46], PMPD micelles [13], and CD147-ODEX-DOX nanoparticles [47], are susceptible to dilution-induced disassembly due to their reliance on noncovalent aggregation, resulting in limited stability scores. BSP-H-DOX micelles [48] exhibit relatively lower drug loading content. CPOF-DOX micelles [32], while effective in drug loading, are prepared using high concentrations of CuBr catalyst, which raises concerns about toxic copper residues and compromises their synthetic benignity score. In contrast, the star copolymer-DOX micelles developed in this work integrate several advantageous features, including unimolecular stability that is independent of concentration, high drug loading capacity, favorable biocompatibility, and effective *in vitro* cytotoxicity. Moreover, the system was synthesized via a metal-free route that avoids the use of transition-metal catalysts, further improving its suitability for biomedical applications. Collectively, these attributes underscore the potential of this system as a synthetically accessible and structurally robust prodrug nanocarrier platform.

## 4. Conclusions

In summary, we report a facile and environmentally benign strategy for constructing pH-responsive DOX-conjugated amphiphilic star copolymers via metal-free ATRP combined with post-functionalization. A hydroxyl-functionalized star copolymer scaffold was first prepared and subsequently modified to introduce pendant benzaldehyde groups, enabling DOX conjugation through imine linkages. This strategy decouples polymer architecture from functionalization, thereby circumventing the need for pre-synthesized customized monomers. Owing to the star-shaped architecture, the resulting prodrug forms stable unimolecular micelles with concentration-independent colloidal stability and pH-triggered drug release behavior. *In vitro* studies further demonstrate effective cellular internalization and a moderated cytotoxic profile toward cancer cells, supporting the utility of this system as a modular polymeric prodrug platform for further development and evaluation.

## Supplementary Material

Supplemental Material

## Data Availability

The original contributions presented in this study are included in the article. Further inquiries can be directed to the corresponding author.
