# Supplementary material for: DOX-conjugated unimolecular micelles from benzaldehyde-functionalized star copolymer via metal-free ATRP for pH-responsive drug delivery
Source: Sci Technol Adv Mater. 2026 Feb 2;27(1):2624889. doi: 10.1080/14686996.2026.2624889 (PMC12973830; doi:10.1080/14686996.2026.2624889)
Supplement: Supplemental Material [file TSTA_A_2624889_SM9068.docx]

Supporting Information

**DOX-Conjugated Unimolecular Micelles from Benzaldehyde-Functionalized Star Copolymer via Metal-Free ATRP for pH-Responsive Drug Delivery**

The molar ratio of HEMA to OEGMA in the star copolymer-OH was calculated according to the following equation:

$\frac{I_{\text{a}}}{I_{\text{h}}}=\frac{2(\text{m}+\text{n})}{3\text{n}}$,

where *I*_a_ and *I*_h_ represent the integrated intensities of the methylene protons in the backbone (a) and the methoxy protons in the OEGMA’s side chain (h) in Figure 1a, respectively; m and n denote the degree of polymerization for HEMA and OEGMA, respectively. Based on this calculation, the HEMA to OEGMA molar ratio was determined to be m: n = 0.9:1.

The conversion ratio of -OH functionality to benzaldehyde group was calculated based on the following equation:

conversion ratio = $\frac{3\text{n}\times I_{\text{k}}}{m\times I_{\text{h}}}$

where *I*_k_ and *I*_h_ represent the integrated intensities of peak k and h in Figure 1b, respectively; m and n denote the degree of polymerization of HEMA and OEGMA in the star copolymer-OH, respectively. According to this equation, the conversion ratio was determined to be 99%.


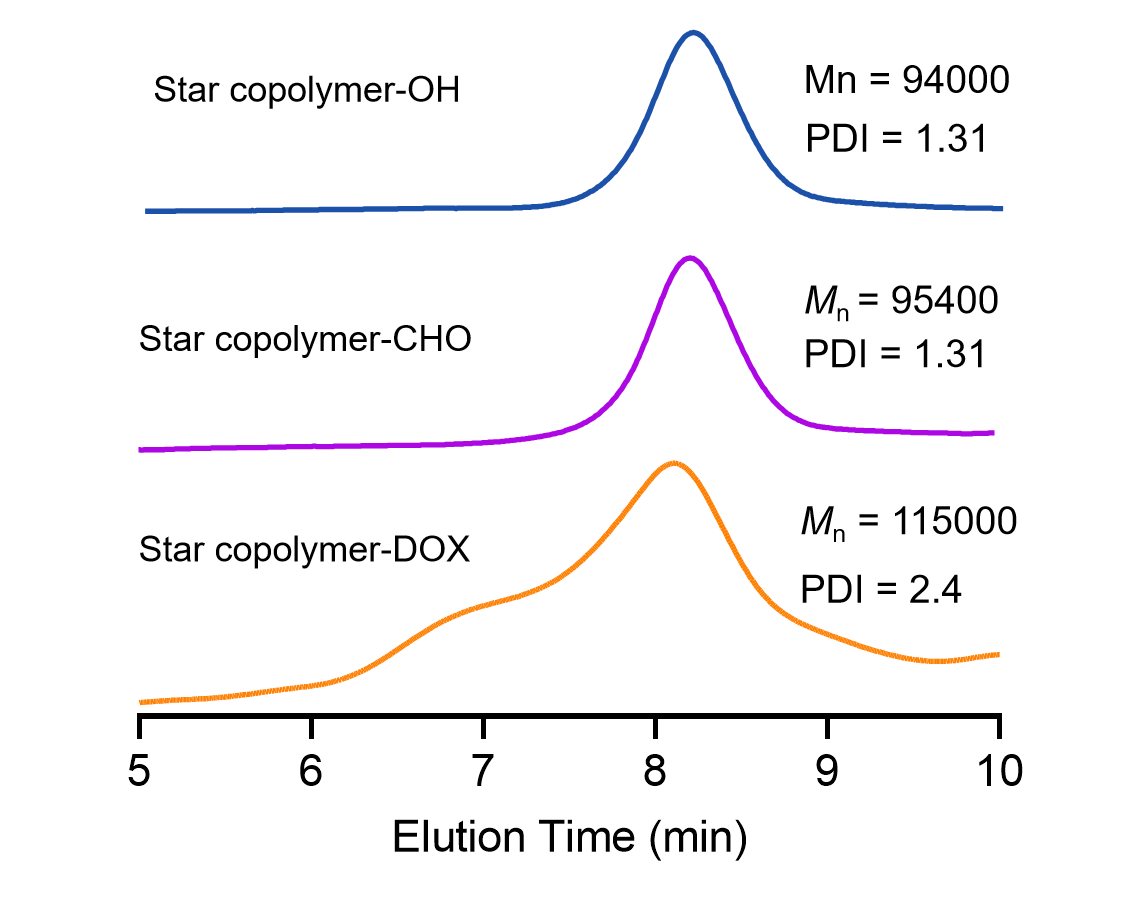


**Figure S1.** GPC curves of star copolymer-OH, star copolymer-CHO, and star copolymer-DOX

GPC analysis was performed in DMF to monitor the molecular characteristics of the star copolymers at different stages of synthesis. The star copolymer-OH exhibited an apparent number-average molecular weight (*M*ₙ) of approximately 94 kDa with a molecular weight distribution (PDI) of 1.31, indicating a controlled metal-free ATRP process from β-CD-Br.

After post-polymerization modification of the hydroxyl groups to benzaldehyde functionalities, the resulting star copolymer-CHO showed a slightly increased apparent *M*ₙ (95.4 kDa) with an unchanged PDI, suggesting that the chemical transformation proceeds without detectable chain coupling or degradation.

Following DOX conjugation, the GPC trace of star copolymer-DOX shifted to shorter retention times and became broader, giving an apparent *M*ₙ of approximately 115 kDa and a PDI of 2.40. This behavior may be associated with drug incorporation and non-ideal GPC effects commonly encountered in drug-conjugated polymer systems.


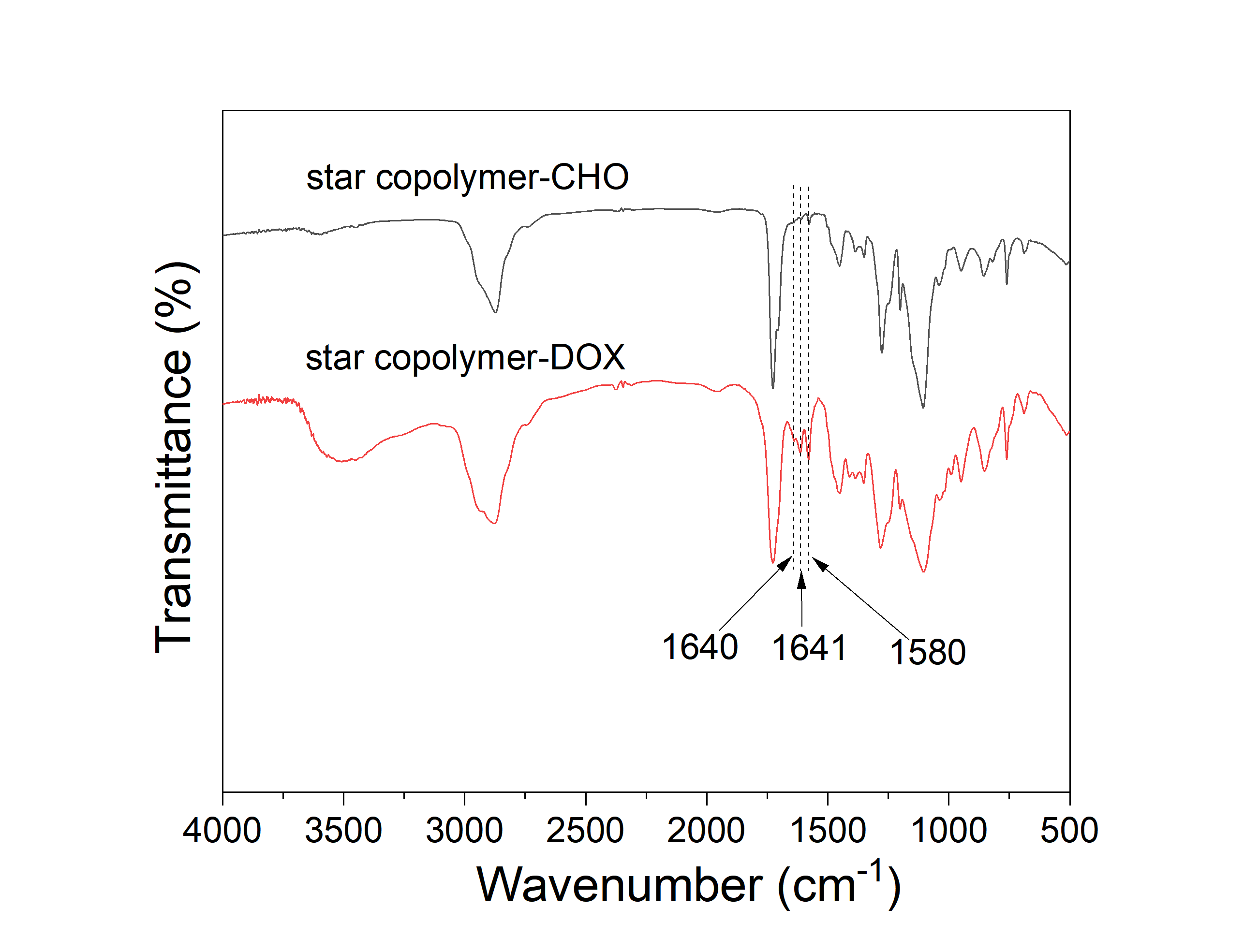


**Figure S2.** FT-IR spectra of the star copolymer-CHO and the star copolymer-DOX

FTIR spectrum of the star copolymer-CHO exhibits a weak absorption around 1580 cm⁻¹, which can be attributed to aromatic ring vibrations associated with the benzaldehyde moieties. After conjugation with DOX, the FTIR spectrum of star copolymer-DOX shows a pronounced enhancement of the band at ~1580 cm⁻¹, together with two newly emerging absorptions at ~1614 and ~1640 cm⁻¹ (Figure S2). The appearance of the band at ~1640 cm⁻¹ is consistent with the C=N stretching vibration typically associated with imine linkages, while the bands in the 1580–1614 cm⁻¹ region likely arise from increased contributions of aromatic and conjugated structures introduced by DOX.


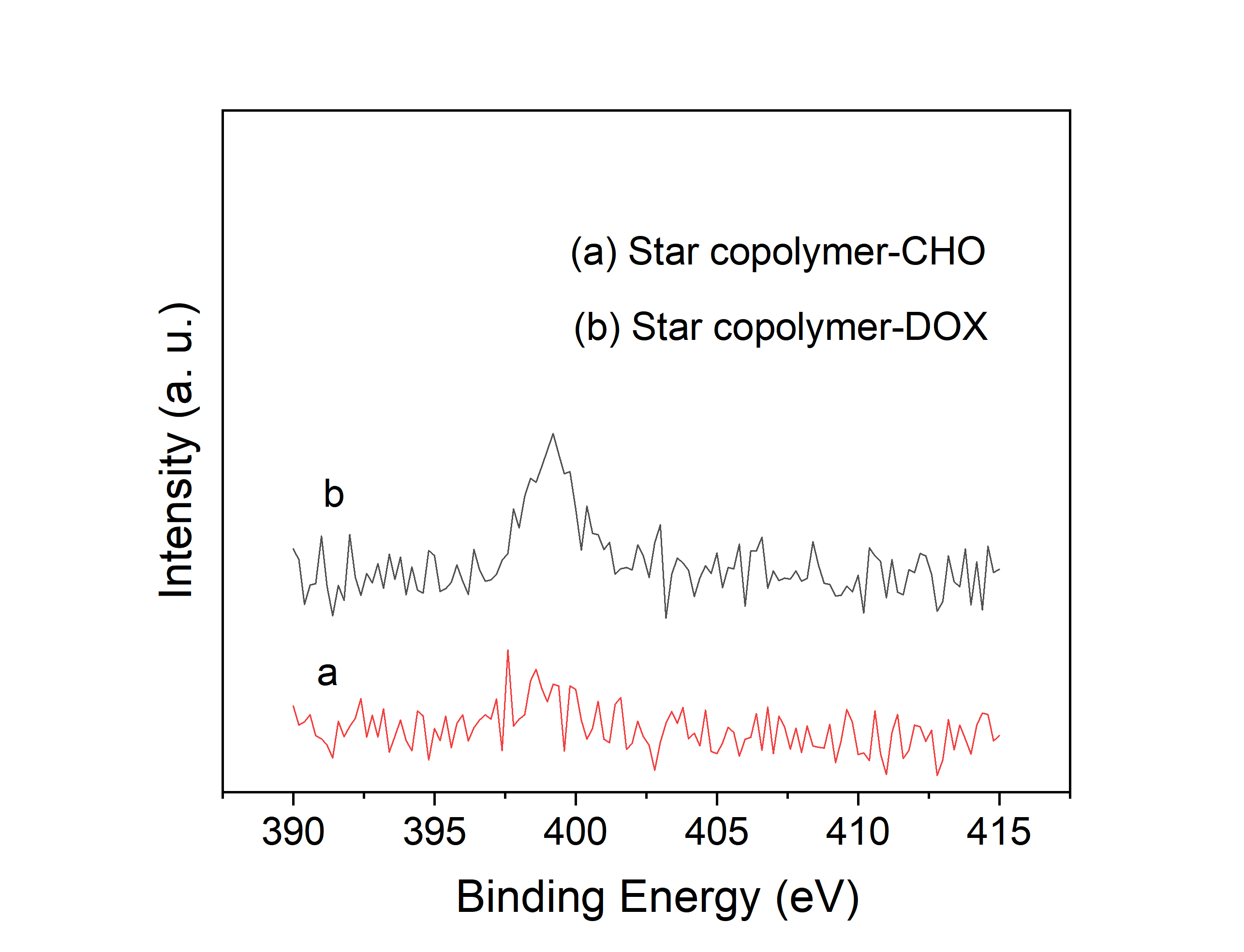


**Figure S3.** High resolution XPS spectra of the star copolymer-CHO and the star copolymer-DOX.

XPS measurements further corroborate the successful incorporation of DOX into the star copolymer. As shown in Figure S3, star copolymer-CHO shows no discernible N_1s_ signal, whereas star copolymer-DOX exhibits a clear N_1s_ peak centered at ~399 eV. This binding energy is characteristic of nitrogen species in imine or amine-related chemical environments. The emergence of the N_1s_ signal in star copolymer-DOX, together with the absence of nitrogen in star copolymer-CHO, confirms the introduction of nitrogen-containing DOX moieties into the polymer. Combined with FTIR and NMR results, the XPS data provide complementary evidence supporting DOX conjugation via imine linkage.


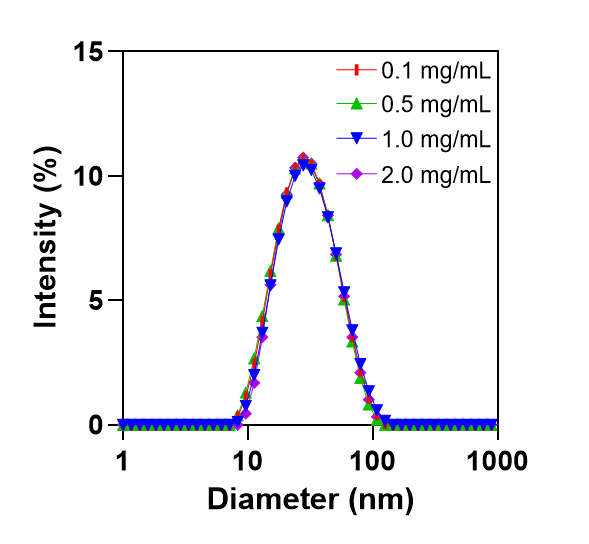


**Figure S4.** DLS data of the star copolymer-DOX micelles measured at different polymer concentrations.


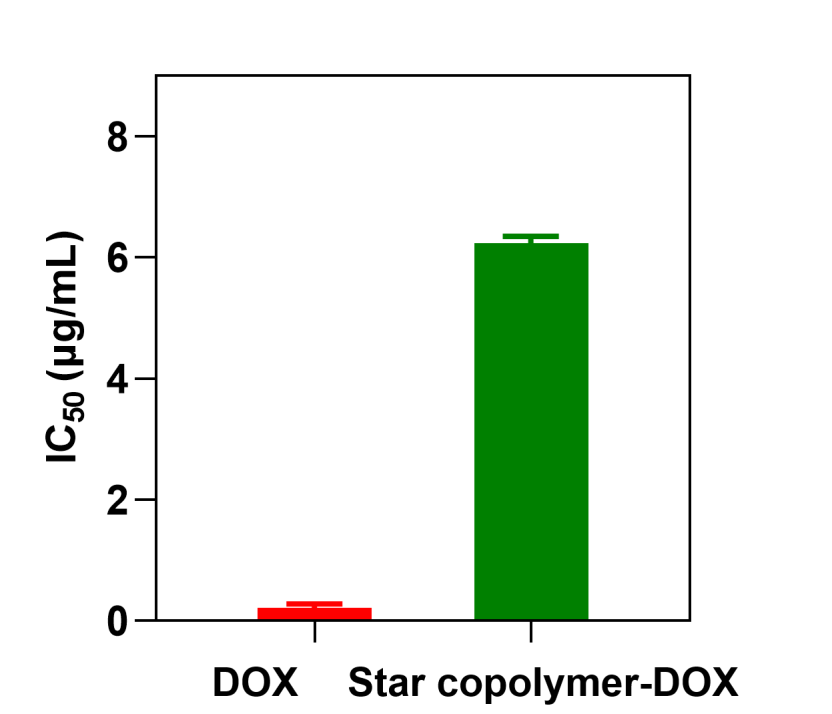


**Figure S5.** The half maximal inhibitory concentration (IC_50_) of free DOX and the star copolymer-DOX micelles against H460 cells, determined from dose-response curves based on DOX-equivalent concentrations. Data are presented as mean ± SD (n = 3).

**Scoring criteria used in Figure 7:**

**Stability:** linear-structured prodrug micelles (score = 0); star-shaped prodrug micelles (score = 1).

**Synthetic benignity**: transition metal-based preparation (score = 0); metal-free preparation (score = 1).

**Drug loading content (DLC)**: DLC > 15% (score = 1); DLC ≤ 15% (score = 0.5).

**Biocompatibility**: cell viability > 95% across all tested concentrations (score = 1).

***In vitro* cytotoxicity**: cancer cell viability suppressed to < 50% within the experimental concentration range (score = 1).
